# HARNESSING THE POWER OF A GERIATRIC EXERCISE NETWORK TO IMPROVE HEALTH: THE VA GEROFIT WAY

**DOI:** 10.1093/geroni/igz038.132

**Published:** 2019-11-08

**Authors:** Katherine S Hall, Janet Prvu-Bettger

**Affiliations:** 1 Durham Veterans Affairs Health Care System, Durham, North Carolina, United States; 2 Duke University School of Medicine, Durham, North Carolina, United States

## Abstract

The Gerofit Consortium is a clinical and research network that naturally evolved from the dissemination of a VA “Best Practice” exercise and health promotion program for older Veterans. Originally developed in the Durham VA, Gerofit has been disseminated to 15 VA Medical Centers using a successful “collaboratory” team network approach. This group has met regularly (biweekly) for five years; starting with four newly disseminated programs in 2014 and adding 2-3 new programs per year since, following a structured implementation process. We have learned that the sum is much greater than the parts. Each program shares important experiences and contributes to development of new program practices aimed at increasing access and adherence to physical activity for Veterans wherever they live, and improving their overall health and wellness. This consortium has served over 7000 Veterans. The first paper describes the impact of overweight/obesity on functional outcomes. The second abstract describes a newly developing use of live video connection from an exercise leader directly to the home for exercise. The third paper provides a case series on the impact of Gerofit on fall prevention. The fourth paper describes the impact of Gerofit on physical function among Veterans with arthritis. The fifth paper describes the impact of Gerofit on Post Traumatic Stress Disorder (PTSD) symptoms among participating Veterans with self-reported PSTD. This symposium highlights the impact of a highly synergistic network working together to improve the lives of older Veterans.

